# Design and experiment of a novel intelligent device suitable for automation vegetable plug seedling

**DOI:** 10.1371/journal.pone.0336844

**Published:** 2025-11-14

**Authors:** Qiu Haifei, Nie Zhike, Chen Xiandong, Lv Bo, Zhang Yifan, Li Feiyang, Zhang Jiajun

**Affiliations:** 1 School of Mechanical Engineering, Xijing University, Xi’an, China; 2 Engineering Research Center of Hydrogen Energy Equipment & Safety Detection, Universities of Shaanxi Province, Xijing University, Xi’an, China; Shahrekord University, IRAN, ISLAMIC REPUBLIC OF

## Abstract

To tackle the challenges of high manual labor intensity and low efficiency in traditional vegetable plug seedling processes, this study developed an automated mechanical device through innovative design, dynamic simulation, single-chip development, and prototype testing. The device integrates precision seeding technology based on the synchronized motion control of ball screws and crank sliders, which improves the qualification rate of plug sowing to 98% by “one hole one seed” sowing, and also minimizes seed waste and reduces planting costs. Automated processes like hole pressing, sowing, soil covering, fertilizing, and watering are achieved through innovative design and functional integration, ensuring efficient and high-quality plug seedling production. Additionally, real-time monitoring of vegetable cultivation parameters, including seed type, quantity, planting depth, and environmental temperature and humidity, is facilitated by wireless WiFi, intelligent screens, and Alibaba Cloud, offering valuable technical insights for the digital and intelligent advancement of new vegetable plug seedling machinery.

## 1. Introduction

Plug seeding, an advanced cultivation technique that emerged in the 1970s, is widely used for producing commercial seedlings of various vegetables and flowers [1]. This method employs soilless materials such as peat and vermiculite as seedling substrates, providing benefits like uniform seed distribution, high survival rates, low production costs, easy storage and transportation, reduced pest and disease spread, and improved seedling quality [2]. Consequently, it is ideal for intensive management and large-scale production in modern agricultural greenhouses.

However, the production process for vegetable plug trays is complex and labor-intensive. Traditional vegetable plug seedling cultivation primarily relies on manual or single-function mechanical sowing, which often makes it difficult to ensure precise planting depth and spacing [3]. Most of the market machinery about plug seeding lacks a comprehensive solution that aligns with standardized production methods and processes [4]. Existing technologies often depend on ‘needle suction’ for precise seeding, necessitating a sophisticated integration of mechanical and control components [5]. This complexity not only makes the system more challenging but also raises research, development, and maintenance costs [6], which is not conducive to the automation and intelligent development of plug seedling technology.

This paper presents a novel intelligent device for manufacturing vegetable plug trays, aimed at enhancing the efficiency and quality of modern vegetable plug seedling production. The feasibility of the design and the effectiveness of the technical solutions have been validated through mechanism analysis, finite element modeling, dynamics simulation, physical prototype production, and experimentation. The work contributes to innovation in vegetable plug-seeding machinery and holds significant potential for practical applications.

## 2. Background and motivation

**Fig 1:**
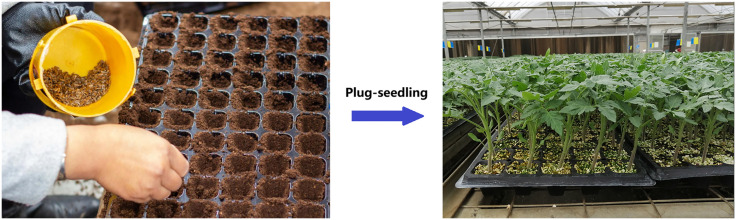
Prefabricated vegetable plug tray.

Vegetable plug seedlings are primarily used in modern agricultural greenhouses. As shown in Fig 1, seeds are planted either manually or with the help of auxiliary equipment into prefabricated trays, which come in various sizes, such as 32, 72, 105, and 128 holes [7]. To reduce planting costs, achieving “one hole one seed” sowing accuracy is essential.

The physical characteristics of various vegetable seeds differ greatly, including their shape, size, planting depth, and moisture needs [8], as illustrated in Table 1. Accurately placing one seed in each hole poses a substantial technical challenge in automating the plug tray production process.

**Table 1:** Physical characteristics of seeds in common vegetable.

Type	Shape	Size (mm)	Sowing depth (cm)	Temperature (°C)	Soil moisture (RH)
**Scallion**	Elliptical/spherical	1.5 ~ 3	1 ~ 2	7	70% ~ 80%
**Cabbage**	Elliptical/spherical	1.8 ~ 2.5	1 ~ 2	20 ~ 25	80% ~ 90%
**Greens**	Spherical	2 ~ 3	1 ~ 2	20 ~ 25	70% ~ 80%
**Spinach**	Elliptical/spherical	2 ~ 3	1 ~ 2	6 ~ 18	70% ~ 80%
**Leek**	Elliptical	0.5 ~ 2	1 ~ 2	10	80% ~ 90%
**Lettuce**	Elliptical	1 ~ 2	5 ~ 10	15 ~ 25	60% ~ 70%
**Radish**	Irregular	2 ~ 4	1 ~ 2	20 ~ 25	60% ~ 80%

## 3. Ideas and methods

### 3.1 Precision sowing

**Fig 2:**
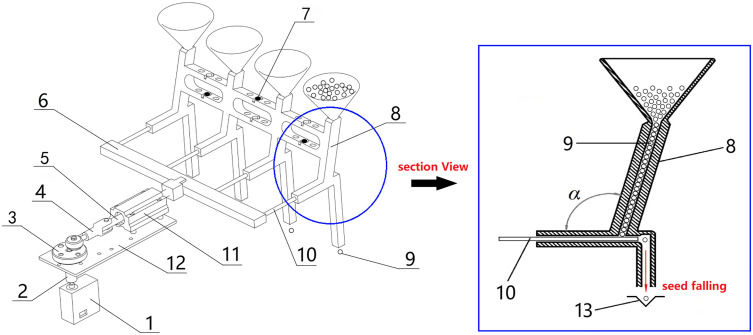
Sowing mechanism and principle. 1-Steering servo, 2-Coupling, 3-Crank, 4-Connecting rod, 5-Slider, 6-Needle holder, 7-Vibrating plate, 8-Hopper, 9-Seeds, 10-Push needle, 11-Support base, 12-Baseplate, 13-Hole.

The device uses a crank-slider mechanism to ensure precise planting with a “single-seed-per-hole” delivery system. As shown in Fig 2, a digitally controlled steering servo motor drives the crank’s rotation through a rigid coupling, converting rotary motion into linear reciprocation via the connecting rod. During each crank revolution (0 → 2π), the push needle dispenses a vegetable seed from the hopper, which then falls into the corresponding hole.

The slider stroke (*S*), a critical parameter governing seed displacement accuracy, is determined by the extreme positions of the crank-connecting rod assembly when collinear [9]. Through geometric analysis of the mechanism’s dead-center positions, the stroke can be calculated as equation (1) [10].


$$S=L_{1}+L_{2}-\left(L_{2}-L_{1}\right)$$ (1)

where *L*_1_ = 7.5 mm (crank radius) and *L*_2_ = 50 mm (connecting rod length). Substituting *L*_1_ and *L*_2_ = 50 mm into equation (1), the substitution yields *S*=15mm, indicating a well-constrained linear displacement range sufficient for reliable seed pushing while maintaining compact mechanism dimensions.

To ensure that vegetable seeds fall one by one, the angle *α* between the center axis of the push hole and the feeding hole is set to an obtuse angle of 110° ± 5°. In addition, three vibrating plates are also installed at different points of the hopper, which can generate intermittent low-frequency vibrations of small amplitude during feeding and pushing(Time interval:1.5s, Vibration frequency:15 Hz, Amplitude:0.05 mm), preventing seeds from clogging at the feeding horn mouth.

### 3.2 Control and cooperation principle

**Fig 3:**
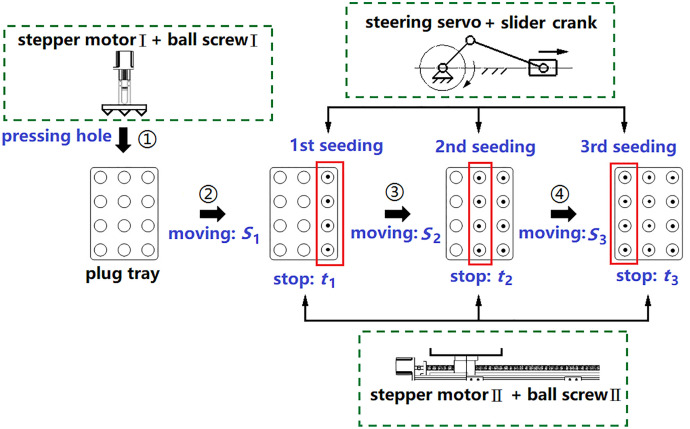
Control principle of pressing holes and sowing.

For accurate planting, precise control of the motion displacement and timing of the hole punching and sowing processes is essential [11].Taking a 12-hole tray as an example, a motion control scheme involving motors, ball screws, and crank sliders is constructed, as shown in Fig 3. When stepper motor I drive ball screw I for hole pressing, stepper motor II simultaneously drives ball screw II toward the sowing end. Upon reaching the set distance *S*_1_, ball screw II pauses for *t*_1_, during which the crank slider performs a reciprocal motion to sow four seeds into four holes. This process is repeated for distances *S*_2_ and *S*_3_ with corresponding pauses *t*_2_ and *t*_3_.

## 4. Structural composition and layout

### 4.1 Functional and implementation

The device integrates essential functions, including hole punching, seed planting, soil covering, fertilization, and watering. Its mechanical and electrical control system comprises components such as ball screws, crank-slider mechanisms, stepper motors, sensors, and single-chip systems. It also offers smart features, including status monitoring and digital displays. For a detailed breakdown of functional divisions and implementation methods, refer to Table 2.

**Table 2:** System function and implementation method.

Function	Implementation method	Remarks
hole pressing	ball screw, stepper motor, cone mold	optional depth(2.8 cm/3.5 cm)
seed sowing	ball screw, crank slider, hopper	one hole one seed
soil covering	brush, adjustment sheet	friction filling
fertilizing, watering	nozzle, nutrient solution	soil moisture and nutrients
status monitoring	display screen, alibaba cloud web	quantity, depth, temperature and humidity
system control	Single-chip, stepper motor	program development, control integration

**Fig 4:**
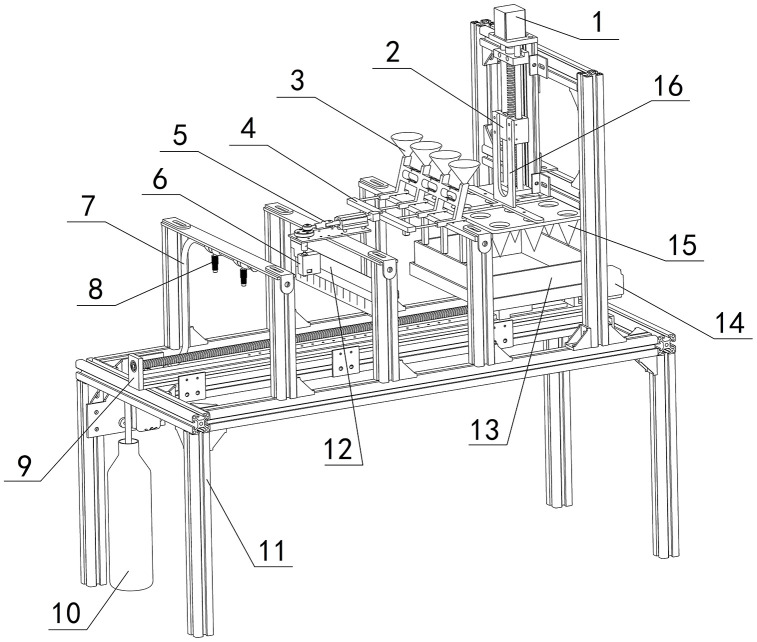
Whole machine structure. 1-Stepper motor Ι, 2-Ball screw Ι, 3-Hopper, 4-Needle holder, 5-Crank slider, 6-Steering servo, 7-Hose, 8-Nozzle, 9-Ball screw Ⅱ, 10-Liquid container, 11-Frame, 12-Brush, 13-Plug tray, 14-Stepper motor Ⅱ, 15-Cone mold, 16-Cross connector.

Fig 4 illustrates the machine’s layout, measuring 1100 × 480 × 1060 mm. Supported by a spatial frame structure, it houses various functional components for electrical control and mechanical operation, including stepper motors, ball screws, steering servos, crank-slider mechanism, brush, nozzles, and hoses, etc.

From Fig 4, this device is divided into four sections: the hole-pressing area, the seeding area, the soil covering area, and the spraying area. Its working principle is as follows: the ball screw I first drives the cone mold-15 to move downward, forming “three rows by four columns” of holes on the soil surface, and then the ball screw II drives the plug tray-13 to perform intermittent linear sliding. Meanwhile, the crank slider-5 cooperates to carry out the precise seed-pushing actions. Subsequently, the plug tray-13 comes into contact with the brush-12 under the action of the ball screw II, and the holes are covered with soil by friction between the brush and the soil surface. Finally, the plug tray enters the spraying area, and the atomizing nozzle-8 sprays the water-soluble fertilizer solution onto the surface of the soil layer.

In addition, the device is equipped with temperature and humidity sensors, as well as a 12864 intelligent screen display, to monitor and present soil conditions and real-time sowing status for the vegetable tray, such as seed name, quantity, etc.

### 4.2 Hole pressing

**Fig 5:**
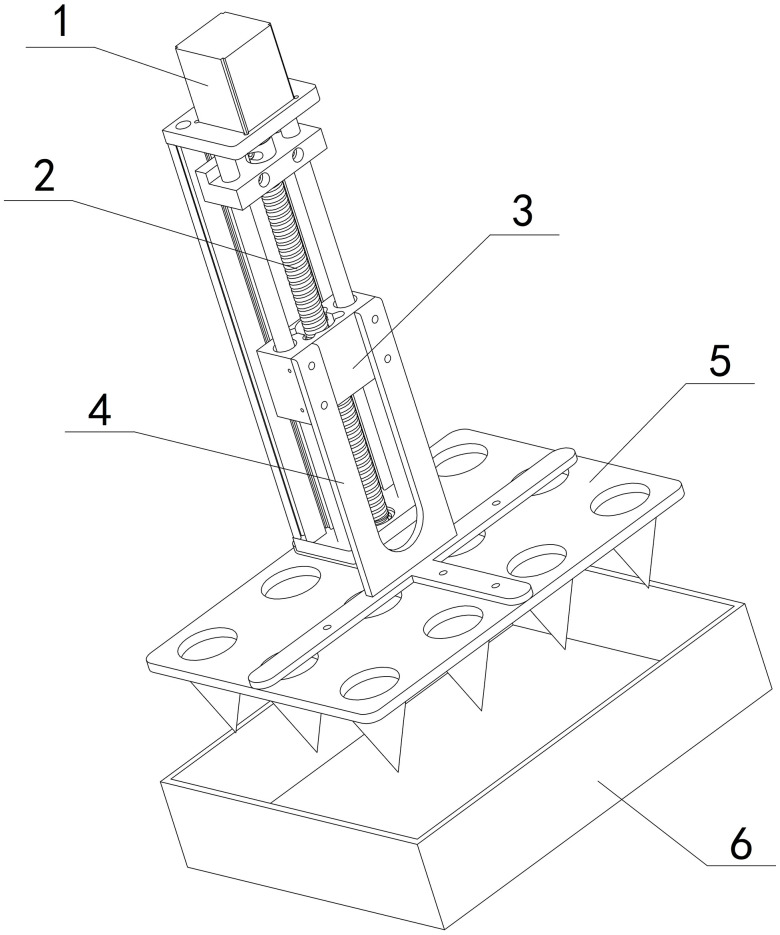
Hole pressing mechanism. 1-Stepper motor Ι, 2-Ball screw Ι, 3-Slide table, 4-Cross connector, 5-Cone mold 6-Plug tray.

To create holes in the soil surface of the plug tray, the cone mold-5 is mounted on the slide table of ball screw I, as depicted in Fig 5. During the hole-pressing process, the stepper motor I rotates the ball screw I forward, moving the slide table-3 and cross connector-4 downward via the cone mold-5 until the conical teeth reach the predetermined depth. After the holes are formed, the stepper motor I is reversed, raising the cone mold-5 back to its initial position.

### 4.3 Plug tray driving

**Fig 6:**
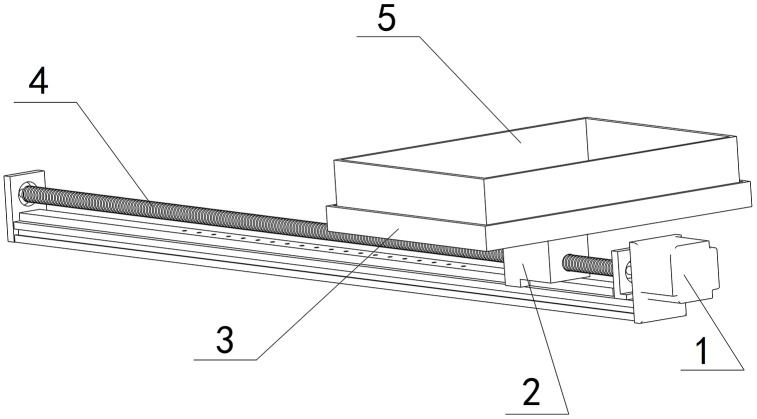
Driving mechanism of the plug tray. 1-Stepper motor II, 2-Slide seat, 3-Supporting tray, 4-Ball screw II, 5-Plug tray.

Concurrently with the reset of ball screw I, ball screw II initiates the linear movement of the plug tray, as depicted in Fig 6. As stepper motor II rotates ball screw II forward, the plug tray-5 advances toward the end of ball screw II, driven by the slide seat-2, and progressively executes the coordinated actions of sowing, soil covering, and spraying via timed control. Upon completion of all processes, remove plug tray-5 for storage and activate stepper motor II in reverse to reset ball screw II.

### 4.4 Soil covering

**Fig 7:**
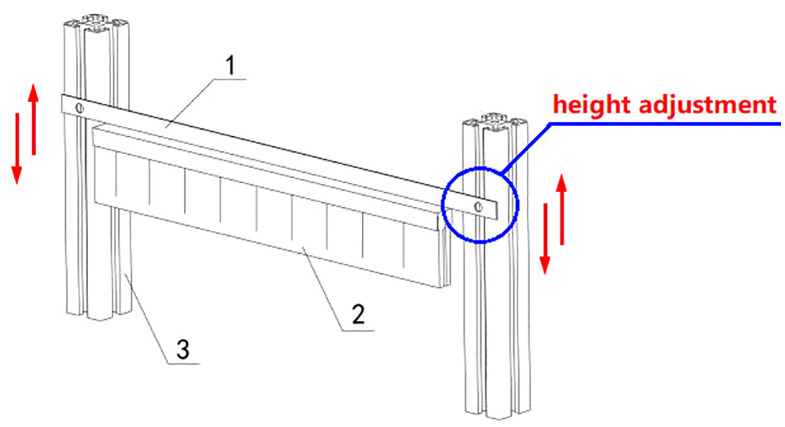
Brush component. 1-Adjustment sheet, 2-Brush 3-Support pillar.

Once the sowing task is complete, the ball screw II moves the plug tray toward the soil covering area. Here, brush-2 contacts the surface soil, using friction to cover the holes, as illustrated in Fig 7. To accommodate the varying planting needs of different vegetables, the thickness of the covering soil can be adjusted by changing the height of adjustment sheet-1 on support pillar-3.

### 4.5 Watering and fertilizing

**Fig 8:**
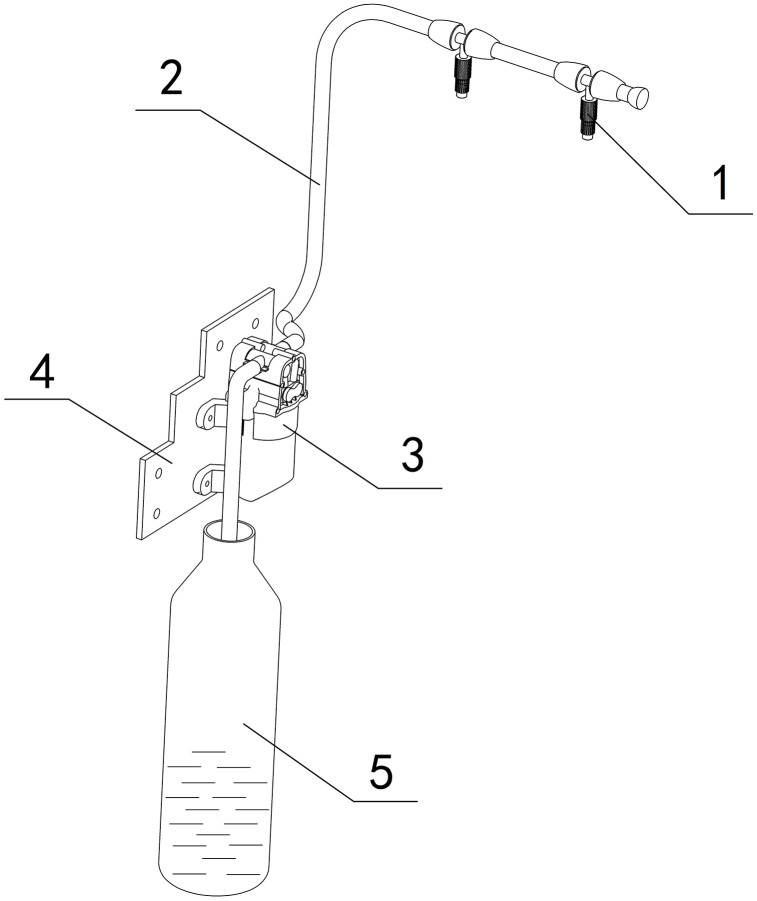
Spray component. 1-Nozzle, 2-Hose, 3-Water pump, 4-Fixed plate, 5-Liquid container.

To meet the requirements for sowing and seedling cultivation of vegetable plug-seedlings, the soil environment must have adequate moisture and nutrients [12]. The device is equipped with an automatic spraying component, as shown in Fig 8. The prepared nutrient solution is preloaded into Liquid Container-5. As the plug tray passes beneath Nozzle-1, the water pump-3 activates, delivering the nutrient solution through Hose-2 to Nozzle-1, and then evenly sprays the nutrient solution on the soil layer, achieving the dual purpose of fertilization and watering.

## 5. Virtual prototype

**Fig 9:**
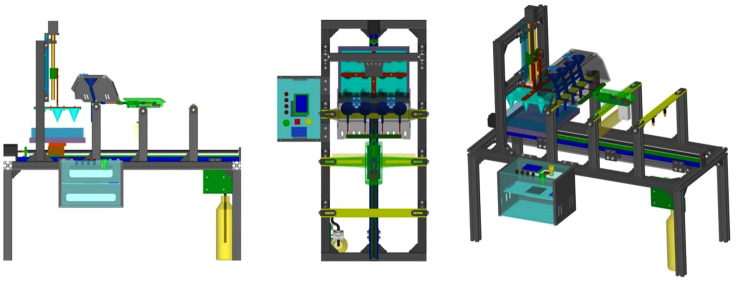
Virtual prototype of the device. (a) Front view (b) Vertical view (c) Axonometric view.

The virtual prototype of the device was created using SolidWorks software, as shown in Fig 9. To improve modeling and assembly efficiency, relevant fasteners like bearings, screws, bolts, and nuts were obtained from the Toolbox design library within SolidWorks. It is crucial to ensure there are no interferences, collisions, or over-constraints in the digital prototype, which is conducive to achieving motion coordination and the desired control [13].

## 6. Static analysis

### 6.1 Finite element modeling

Define boundary conditions based on actual assembly relationships and convert concentrated forces on each component into equivalent static loads [14]. Conduct a finite element static analysis on key load-bearing components, such as the cross connector, cone mold, and plug tray, using the Workbench environment.

Perform a finite element static analysis on critical load-bearing components, including the cross connector, cone mold, and plug tray, within the Workbench environment. Relevant data is provided in Table 3: *F* represents the concentrated load, *S* denotes the bearing area, *P* indicates the equivalent pressure, *σ*_max_ signifies the maximum static stress, *N*_e_ is the number of elements, and *N*_n_ is the number of nodes.

**Table 3:** Data of finite element analysis.

Parts	*F*/N	*S*/m^2^	*P*/Pa	*σ*_max/_MPa	*N*_e_	*N*_n_
**Cross connector**	5.2	0.01	520	0.93	20145	39040
**Cone mold**	1.86	0.028	66.4	0.07	64858	124950
**Plug tray**	40	0.07	571.4	0.0004	39640	77433

**Fig 10:**
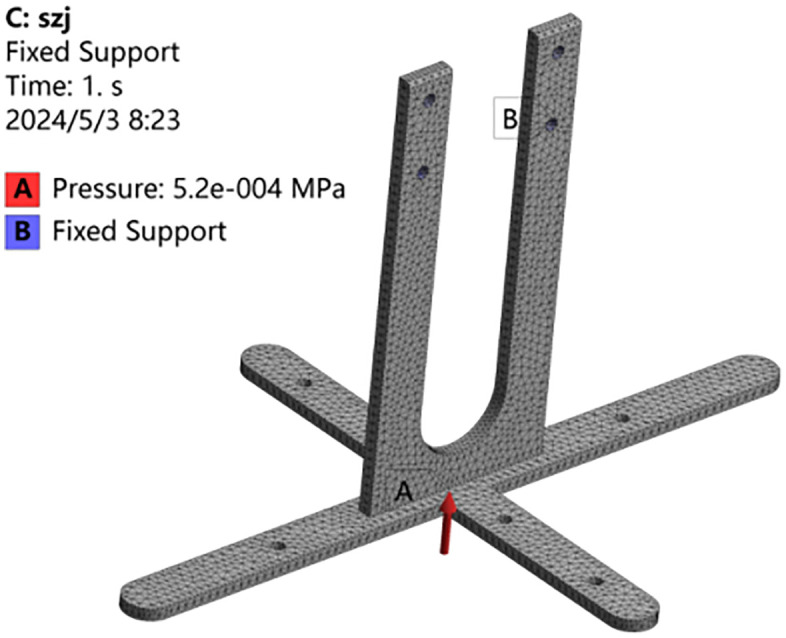
Mesh model of cross connector.

Using the cross connector as a case study, a 20-node hexahedral element (Solid186) and a 10-node tetrahedral element (Solid187) were employed for structural discretization, as shown in Fig 10. The finite element mesh model consists of a total of 20145 elements and 39040 nodes. During the vertical motion of the holes pressing, the bottom section of the cross connector experiences a reverse pressure, as indicated by the red arrow in Fig 10.

### 6.2 Von-Mises stress

**Fig 11:**
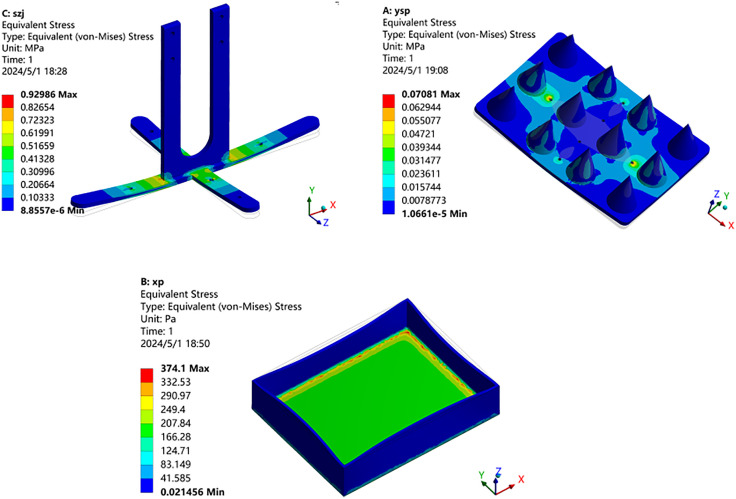
Stress nephogram. (a) Cross connector (b) Cone mold (c) Plug tray.

Stress analysis results for the cross connector, cone mold, and plug tray were obtained through Workbench post-processing, as illustrated in Fig 11. By analyzing the Von-Mises stress nephogram, it is evident that the maximum stress in the cross connector occurs in the transition area between the U-shaped plate and the cross plate, approximately 0.93MPa, as depicted in Fig 11(a). For the cone mold, the highest stress is found at the connection holes along its length, approximately 0.071MPa, as illustrated in Fig 11(b). Conversely, the stress above the plug tray is relatively low, primarily concentrated in the bottom edge area, reaching about 374.1 Pa, as shown in Fig 11(c).

The cross connector, made from iron, has a yield strength of approximately 250MPa to 550MPa. The cone mold and plug tray, crafted from PLA, have a yield strength of around 50MPa. Finite element analysis reveals that the maximum static stress(*σ*_max_) of the cross connector, cone mold, and plug tray is significantly lower than the yield strength[*δ*_limit_] of their respective materials. Based on the maximum Von-Mises stress criterion, as indicated by Equation (2) [15], the static stress analysis satisfies the strength design requirements.


$$\delta_{Von\;Mises}\leq \left[\delta_{limit}\right]$$ (2)

**Fig 12:**
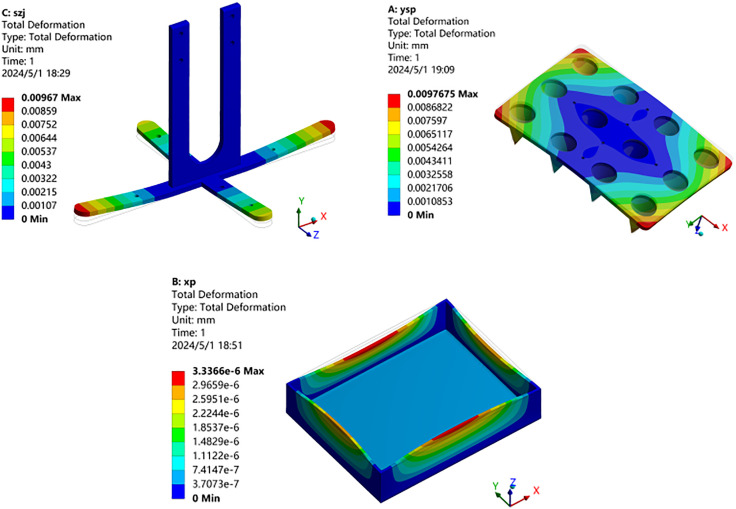
Deformation nephogram. (a) Cross connector (b) Cone mold (c) Plug tray.

From the deformation results shown in Fig 12, it can be observed that the maximum deformation of the cross connector is primarily located at the end of the cross plate, approximately 9.7μm; the maximum deformation of the cone mold mainly appears in the corner area, approximately 9.8μm; and the maximum deformation of the plug tray primarily occurs at the center of the edge of the upright plate in the length direction, approximately 0.003μm. In summary, the maximum deformations of these three components are all in the micrometer range, and the amount of deformation is minimal, which will not have a destructive impact on the structural rigidity.

## 7. CFD numerical simulation

### 7.1 Mesh division of flow field

**Fig 13:**
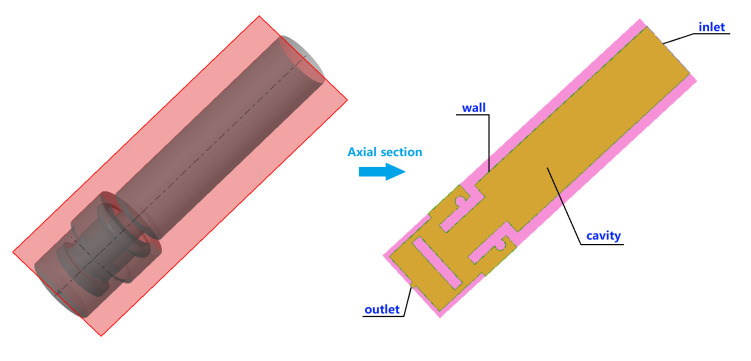
Cavity structure of nozzle.

**Fig 14:**
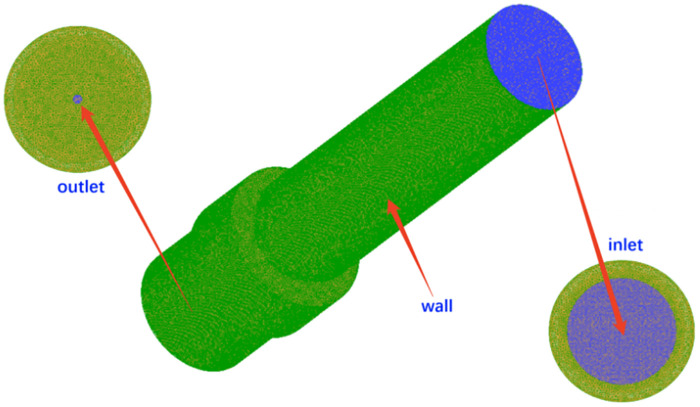
Flow field mesh of nozzle cavity.

Using feature editing and topology reconstruction in ICEM CFD software, a 3D model suitable for CFD simulation calculations was created, as shown in Fig 13. The light yellow area indicates the axial cross-section of the nozzle cavity. A non-structural mesh was employed to discretize the computational domain of the nozzle cavity fluid, as depicted in Fig 14. The flow field mesh division resulted in 5594236 elements and 947789 nodes.

**Fig 15:**
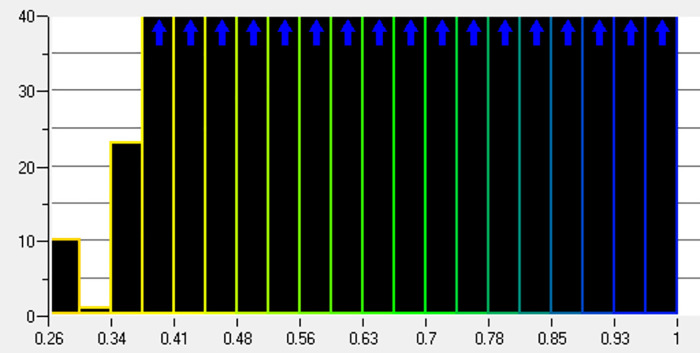
Quality analysis of flow field mesh.

To improve the accuracy of numerical simulations and the reliability of analysis results, mesh refinement was applied to the inlet and outlet areas of the nozzle flow field and specific regions on the wall [16]. The quality analysis of the mesh division as illustrated in Fig 15. The majority of element sizes range from 0.3 mm to 1 mm. However, due to local mesh refinement, some elements are between 0 and 0.3 mm. Notably, there were no negative volume elements in the flow field, indicating excellent mesh quality.

### 7.2 Residual monitoring curve

To accurately assess the atomization spray state of the nozzle, a single-phase steady-state turbulent physical model based on the *k*-*ε* equation was established using FLUENT software [17]. The nozzle operates at a working pressure of 6 kg, with a spray rate ranging from 20 to 260CC/min. The inlet diameter measures 6 mm, while the outlet diameter is 0.5 mm. The inlet pressure is approximately 2.12MPa, and the external environment is maintained at one standard atmospheric pressure (101.325KPa).

**Fig 16:**
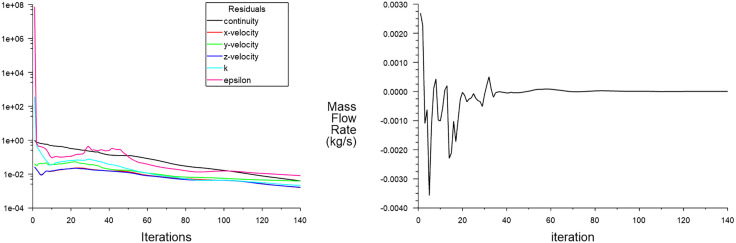
CFD iterative calculation curve. (a) Residual iteration curve (b) Mass flow difference curve.

Based on the initialization of the internal airflow field, a simple algorithm, standard wall function, and second-order upwind scheme were employed to solve the nozzle’s internal flow field using pressure-velocity coupling [18]. As shown in Fig 16, after 140 iterative steps, the residual iteration curve (see Fig 16(a)) and the mass flow difference curve (see Fig 16(b)) stabilized, indicating that the CFD numerical simulation results were converged.

### 7.3 Pressure field and velocity field

**Fig 17:**
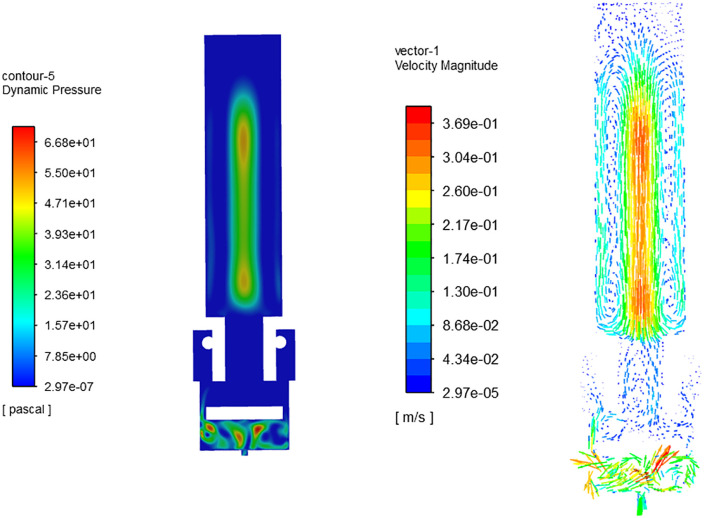
Axial cross-sectional flow field of the nozzle. (a) Pressure field (b) Velocity vector field.

The pressure and velocity vector fields of the nozzle’s axial section were extracted via FLUENT post-processing, as illustrated in Fig 17. The comparison of the flow field distribution reveals that the pressure and velocity fields in the central region of the upper pipeline within the nozzle are significantly higher, with a maximum pressure of about 66.8 Pa and a maximum velocity of approximately 0.37m/s.

In Fig 17(a) and 17(b), near the lower end of the nozzle at the outlet, both the pressure field and velocity vector field exhibit turbulent distribution, which indicates that when water exits through the lower outlet, the pressure within the nozzle cavity is rapidly released, leading to a significant change in the adjacent fluid, thus producing an atomization effect. This aligns with the actual working state of the nozzle.

**Fig 18:**
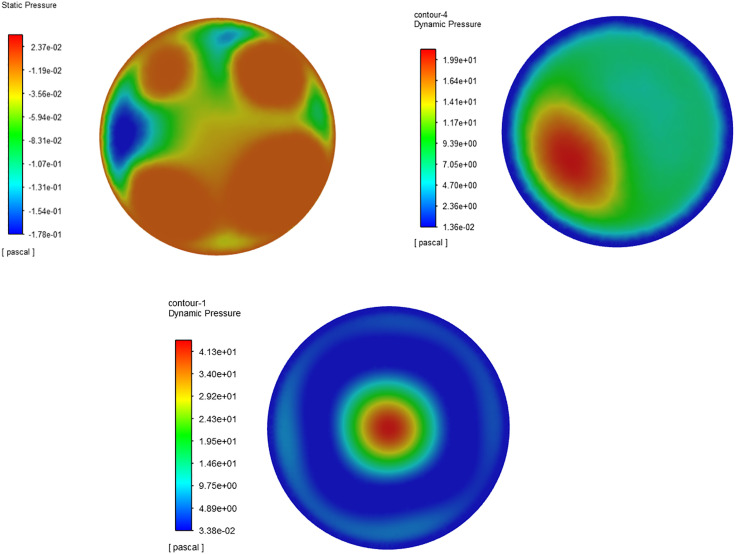
Radial pressure field. (a) Inlet (b) Outlet (c) Radial section of upper pipeline.

The radial pressure nephogram indicates significant changes in pressure distribution at the nozzle’s inlet, with a peak pressure of approximately 237 Pa, as shown in Fig 18(a). In contrast, the outlet’s pressure distribution is more uniform, with a maximum pressure of about 19.9 Pa, as depicted in Fig 18(b). In the radial section of the upper pipeline, the pressure field decreases gradually from the central area to the edge along the radial direction, as illustrated in Fig 18(c). The maximum pressure in this section is around 41.3 Pa, indicating a pronounced high-pressure zone at the center of the upper pipeline. This pressure differential facilitates rapid downward water flow along the axial center.

**Fig 19:**
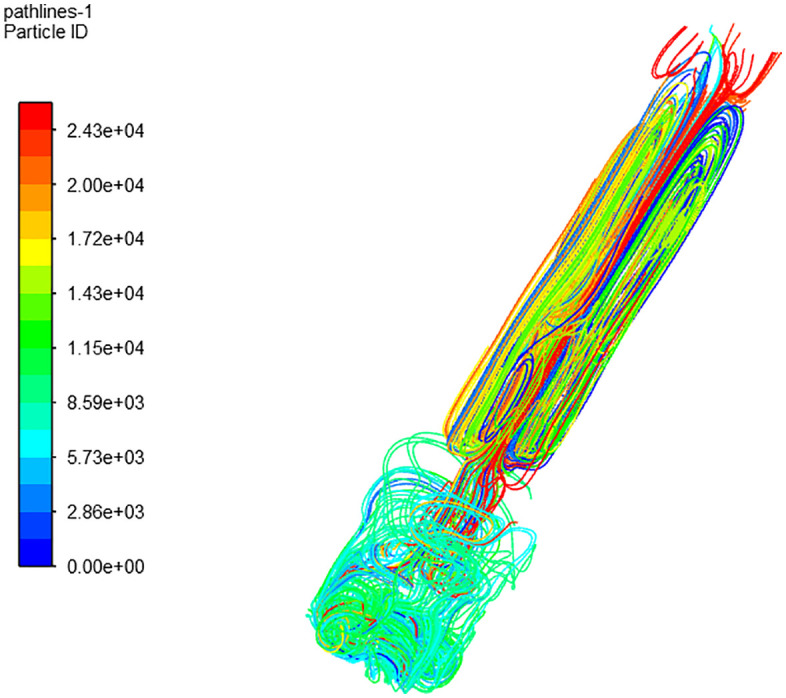
Axial section streamline trajectory.

Using FLUENT post-processing, extract the streamline trajectory of the longitudinal section within the nozzle, as depicted in Fig 19. It is evident that upon entering the nozzle cavity from the inlet, the water flow follows a regular pattern from top to bottom. However, the local flow trajectory exhibits turbulence, aligning with the pressure and velocity field distribution within the nozzle cavity.

## 8. Experiment

### 8.1 Physical prototype

**Fig 20:**
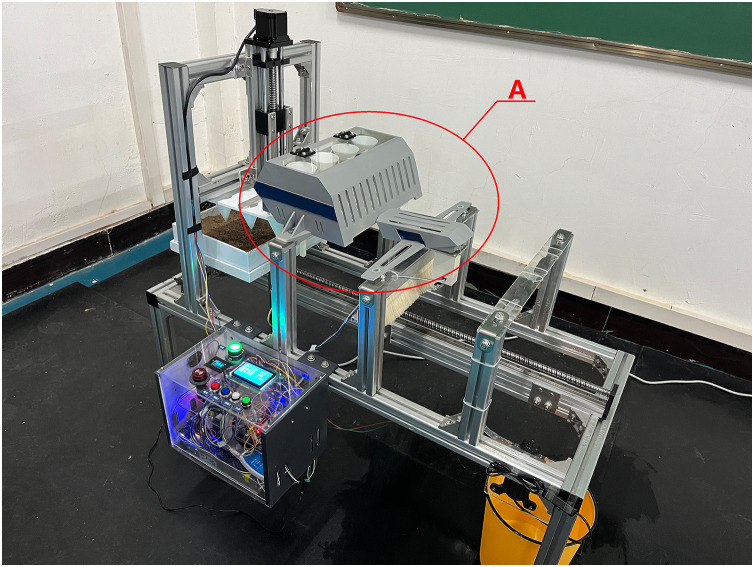
Experimental prototype.

**Fig 21:**
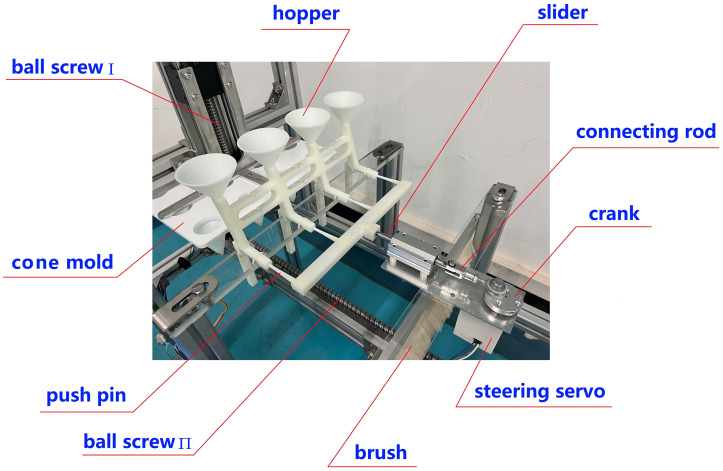
Internal structure of area A.

Assemble each mechanical functional module and electrical control unit according to the overall layout shown in Fig 20. The physical prototype’s frame is made from industrial aluminum profiles, while non-standard components like the cone mold, hopper, and body shell are created from PLA plastic or photosensitive resin through 3D printing. Components that require high strength, such as the cross connector and corner pieces, are manufactured using machining or welding processes. The internal structure of area A is illustrated in Fig 21.

### 8.2 Control system

The primary electronic components of the control system are listed in Table 4. The electromechanical control system utilizes the STM32 single-chip. The entire machine is powered by a 24V5A Mingwei power supply (D-120A). Stepper motor I is driven by the FUYU module, stepper motor II by the TB6600, and the crank slider by a 20 kg steering servo. The water pump’s pumping and atomization solution spraying are controlled via a 5V relay and an inching switch.

**Table 4:** Electronic components of control system.

Order	Components	Specification and model	Number
**1**	Development board	STM32ZET6	1
**2**	Minimum system board	STM32C8T6	1
**3**	Stepper motor driver Ι	FMDD50D40NOM	1
**4**	Stepper motor driver Ⅱ	TB6600	2
**5**	Electric relay	5V	2
**6**	Temperature sensor	DTH11	1
**7**	Humidity sensor	3.3V ~ 12V	1
**8**	Display screen driver	SSD1306	1
**9**	Electronic screen	FG12864F	1
**10**	Wireless module	ESP-8266	1
**11**	Bus servo motor	Torque: 20 kg	1
**12**	Inching switch	5V	5
**13**	Switch power	D-120A/24V 5A	1
**14**	Flash buzzer	12V	1

### 8.3 Functional experiment

**Fig 22:**
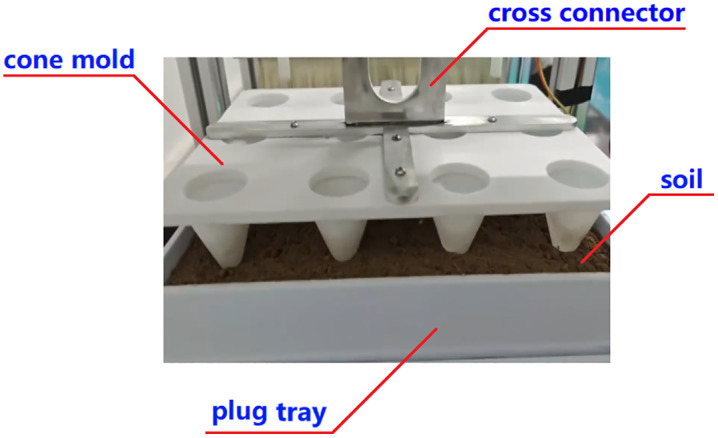
Hole pressing.

**Fig 23:**
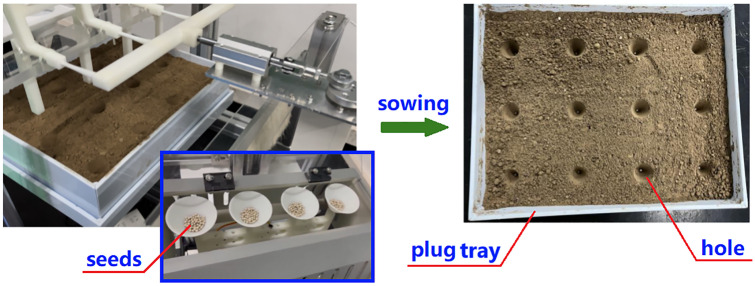
“One hole one seed” sowing.

Press the start button to initialize the machine, then choose a sowing depth of either 2.8 cm or 3.5 cm to begin the hole pressing process. This action drives ball screw I downward to press the hole, as shown in Fig 22. When the cone mold reaches the desired depth, the screw retracts to its initial position. At the same time, ball screw II moves the plug tray toward the sowing end, working with the crank slider to perform the sowing operation according to the scheduled timing, as illustrated in Fig 23.

**Fig 24:**
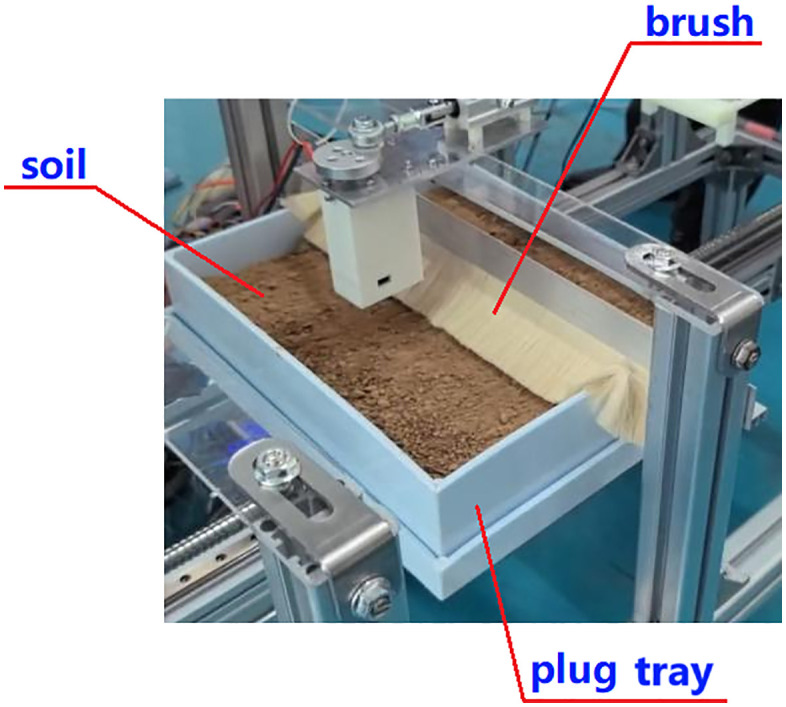
Covering holes with soil.

**Fig 25:**
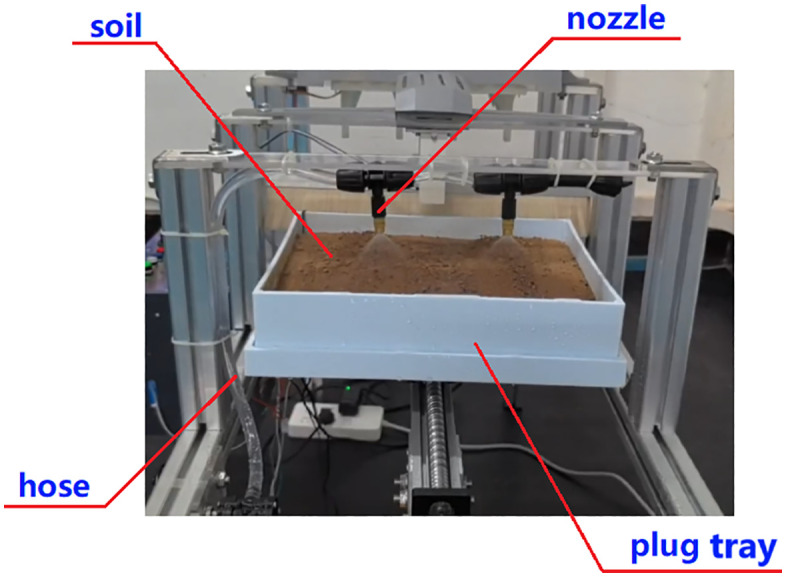
Spray solution.

Following this, the plug tray advances via the ball screw II into the soil covering area and subsequently the spraying area, as depicted in Figs 24 and 25. Once the solution spraying is complete, the plug tray stops moving. At this point, remove the plug tray for backup and press the reset button to reset the ball screw II, preparing for the next sowing.

The sowing experiment, conducted at two depth settings (2.8 cm or 3.5 cm), showed that the device can sow one plug tray in approximately 25 seconds, with a qualification rate exceeding 98%. The device demonstrates stable performance and reliable operation, efficiently handling tasks such as hole pressing, sowing, soil covering, fertilization, and watering with a single click. The experimental results align with the design expectations.

### 8.4 Intelligent monitoring

**Fig 26:**
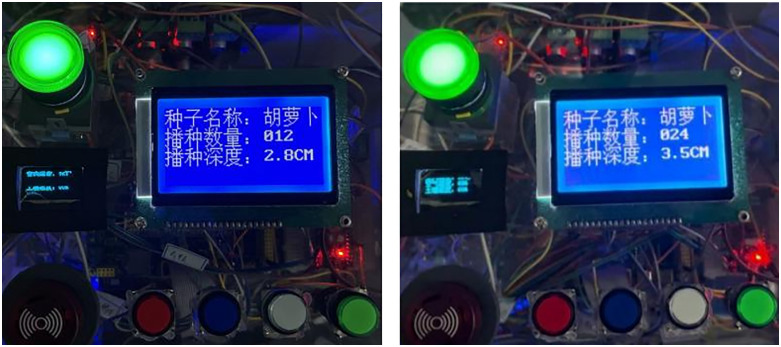
Sowing status display. (a) Sowing depth 1(2.8 cm) (b) Sowing depth 2(3.5 cm).

To accurately monitor the operating status of the experimental prototype, the 12864 intelligent screen display is used to track the sowing process in real-time, as shown in Fig 26. Each time the crank slider and ball screw II complete a coordinated motion, the number of seed orders increases accordingly. Consequently, when using a 12-hole plug tray with three rows and four columns, the cumulative number of seeds per playback cycle is 4.

Due to the varying physical characteristics of vegetable seeds, different vegetables require different sowing depths [19]. To accommodate the diverse planting needs of vegetable plug-seedlings, the experimental prototype includes two hole-pressing depths: 2.8 cm and 3.5 cm, as shown in Fig 26(a) and 26(b). To accommodate the diverse planting needs of vegetable plug-seedlings, the experimental prototype includes two hole-pressing depths: 2.8 cm and 3.5 cm, as illustrated in Fig 26(a) and 26(b). The appropriate planting depth should be selected based on the specific conditions required by the vegetable seeds.

**Fig 27:**
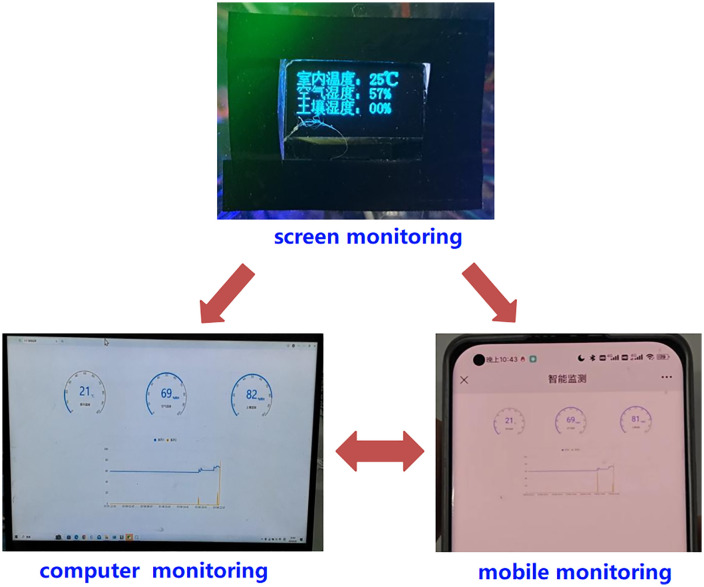
Environmental monitoring on Alibaba Cloud Web.

Utilizing the Keil5 standard library, Alibaba Cloud IoT services, and WiFi data transmission, a temperature and humidity monitoring system for vegetable plug-seedling environments was developed. This system integrates the ESP-01S module, STM32F103VET6 microcontroller, DHT11 temperature sensor, and soil moisture sensor, as depicted in Fig 27. Through dashboard values and real-time curve changes, it allows remote monitoring of soil moisture levels and environmental temperature and humidity within the vegetable plug trays via computer or mobile phone, which could provide robust support for temperature and humidity regulation and watering operations.

## 9. Ethics statement

We declare that this study was conducted in compliance with the ethical principles of the Declaration of Helsinki and the requirements of our Institutional Review Board. All data generated during the study were securely stored and accessible only to authorized personnel. No animals were used in this study, and all experimental procedures followed the relevant national and international guidelines for animal welfare. We certify that this research study fully complies with the ethical principles of the Declaration of Helsinki.

## 10. Conclusions

Most of the existing vegetable plug seedling equipment adopts the “needle suction” on-demand operation, that is, through negative pressure adsorption and positive pressure blowing to complete the precise placement of a single seed. This plug cultivation mode generally has a more complex pneumatic layout and electromechanical composition. A large number of production practices show that the pass rate of “needle suction” on-demand sowing is generally lower than 95%. When large quantities of high-speed operations are carried out (>1000/h), the rate of missed sowing will increase significantly. The waste caused by this will not only reduce the utilization rate of the plug tray but also directly increase the comprehensive cost of vegetable seedlings.

This paper introduces a mechanical vegetable plug-seeding device tailored for modern agricultural greenhouses. It employs a novel precision sowing method based on ‘mechanical cooperation,’ ensuring ‘one seed per hole’ during sowing and ‘one plant per hole’ during seedling development. Compared to needle suction sowing, this sowing mode effectively reduces seed wastage, lowers agricultural planting costs, and significantly enhances the reliability and success rate of plug cultivation. Additionally, the device integrates multiple functions, including hole pressing, sowing, soil covering, fertilizing, and watering, along with practical features like depth selection, intelligent screen display, and environmental monitoring. These features boost production efficiency and digital management in contemporary vegetable plug-seeding practices.

## 11. Future work

Despite advancements, there is still significant potential for improvement in the automatic feeding at the front end and unloading at the back end of the plug-seeding process due to factors like site limitations, experimental conditions, and associated design and production costs. In the future, our team intends to strategically allocate resources, overcome these challenges, and develop plans to integrate conveyor belts, robotic arms, and PLC control systems to address the automatic loading and unloading of plug trays.

Besides, machine vision technology will be utilized to monitor the seeding process, ensuring the qualified rate of sowing in the hole, which will expected to significantly enhance the efficiency of plug seedling cultivation and the reliability of precise seeding, effectively reduce the overall cost of vegetable plug seedling cultivation, and contribute to cost reduction, efficiency improvement, and high-quality development in the modern agricultural vegetable seedling cultivation industry. Finally, we anticipate developing a fully automated production line for modern vegetable plug-seedlings, which will improve the efficiency and productivity of the entire process.

## Supporting information

S1 File CFD simulation. (ZIP)

S2 File Finite element analysis. (ZIP)
